# The Effect of Sports and Recreational Programs on the Well-Being of Children and Adolescents Without Parental Care: A Systematic Review

**DOI:** 10.11621/pir.2025.0401

**Published:** 2025-12-01

**Authors:** Petar M. Spaić, Ivana M. Milovanović, Radenko M. Matić, Nemanja Lakićević, Nebojša Maksimović, Patrik Drid

**Affiliations:** a University of Novi Sad, Serbia; b Lomonosov Moscow State University, Russia; c Federal Scientific Center for Psychological and Interdisciplinary Research, Moscow, Russia

**Keywords:** physical education, sports, children and adolescents without parental care, orphans and vulnerable children

## Abstract

**Background.:**

Children and adolescents without parental care due to parental loss resulting from neglect, abandonment, abuse, institutionalization, or other social circumstances represent a vulnerable population. They face physical and mental health challenges, worsened by the absence of stable support systems. Understanding the benefit that sports and recreational activities can have on improving their well-being is essential.

**Objective.:**

This paper synthesizes existing scientific evidence of the effects of sports and recreational programs on the physical and psychosocial well-being of children and adolescents without parental care, with a particular focus on their speciic circumstances and vulnerabilities.

**Design.:**

Eligibility criteria included studies that examined interventions aimed at improving physical health, anthropometric development, and psychosocial well-being among orphans and vulnerable children (OVC). The focus was on OVC af ected by parental loss, abandonment, neglect, abuse, or other social circumstances. All studies were assessed using the Mixed Methods Appraisal Tool (MMAT).

**Results.:**

Six studies met inclusion criteria, with 331 participants aged 5-21 years. The interventions varied in duration, type, frequency, and intensity, lasting from 3 months to 4 years with training from several times a week to continuous participation. Sessions ranged from 10 minutes to 2 hours. Some programs included personal development, motivational stories, arts, and cultural activities. Interventions improved physical fitness, reduced aggression and risky behaviors, and enhanced self-confidence, social interaction, and academic performance.

**Conclusion.:**

Included studies demonstrated the positive impact of sports and recreational programs on physical and psychosocial well-being, including improvements in physical itness, self-conidence, inclusion, reduction of high-risk behaviors, and academic performance.

## Introduction

Orphans and vulnerable children (OVC) represent one of the most at-risk groups in society, facing challenges that require attention and support on a worldwide scale. These children, deprived of basic parental protection and care, often encounter numerous psychological, emotional, and social challenges. (Fielding-Miller et al., 2015). The absence of parental support increases their vulnerability to institutional disadvantages and long-term negative outcomes, while also reducing their chances for adequate education and vertical mobility on the socio-economic ladder (Boutayeb, 2009). According to the UN Guidelines for Alternative Care of Children (United Nations, 2009), children without parental care are those who do not live with at least one parent, regardless of the reasons or circumstances.

Various studies have proposed and implemented community-based interventions for children and adolescents at risk in different parts of the world (Schenk, 2009). Th ese interventions often include psychosocial support, health services, educational programs, and social inclusion activities, with a particular emphasis on improving overall well-being and resilience. This broad scientific deliberation has provided information that forms the basis for inclusive policies and support programs that enable necessary assistance to diferent marginalized groups of children and adolescents (Bello & Pillay, 2019). At the same time, effective provision of assistance and useful program evaluation can be hindered by inadequate attention to the heterogeneity of risks and needs of children. Identifying the constant gaps between needs and available services requires the ability to distinguish the circumstances in which children find themselves, in order to identify their specific vulnerabilities and provide an appropriate combination of targeted interventions (Goldberg & Short, 2016). Several factors can result in a child being characterized as vulnerable, including the financial difficulties of the parents, ongoing family conflicts, malnutrition, rejection by parents or guardians, or abandonment due to the death of one or both parents as a result of natural death or illnesses such as HIV (Bello & Pillay, 2019; UNICEF, 2017a). According to Pritchard et al. (2016) and Bettmann et al. (2015), the increased costs of social and health care for OVC represent a signiicant global economic and health problem. Neglecting the basic needs of at-risk children and adolescents today leads to substantial and costly long-term consequences for a country’s economy (Loren, 2012). The study by Prywes et al. (2004) highlights that in Eritrea and Benin (countries in East and West Africa), the annual cost of late intervention for social and health care per child from a vulnerable population exceeds 2.000 dollars, while early intervention, assistance, and prevention cost less than 100 dollars per child. Th erefore, it is important to predict costs and implement preventive actions through tailored interventions for different types of vulnerabilities per target population (Alhazmi et al., 2021).

To date, to our knowledge, few studies have thoroughly examined the impact on the mental health and anthropometric status of OVC through the intervention of sports and recreation in addressing key issues, such as OVC self-confidence and self-esteem, adapted inclusive programs, motivation and adaptive potential of OVC in physical education, levels of physical activity, and energy expenditure assessed using quantitative and qualitative methods. Therefore, the current body of knowledge on this matter needs to be comprehensively summarized. However, current research remains fragmented, lacking a systematic synthesis of findings on the effects of sports and recreational interventions on the physical health and psychosocial well-being of OVC populations. An additional issue in existing literature concerns the conceptual and empirical ambiguity in deining the study population. Speciically, most available studies do not make a clear distinction between children who are orphans and those who are institutionally cared for or abandoned for other reasons. Consequently, the factors that led to the absence of parental care and the child’s placement in residential institutions have not been fully considered. Moreover, the variability in the type and duration of placement is rarely addressed — that is, whether it involves short-term/transitional or long-term/multi-year institutional care. Therefore, both the length of stay in the institution and the circumstances leading to the absence of parental care have not been adequately considered when defining the research sample.

Due to these gaps, this study specifically focuses on children and adolescents without parental care, *i.e*., those who do not live with at least one parent due to loss, abandonment, institutionalization, or placement in foster care. This includes orphans, children who have lost one or both parents, as well as children placed in residential institutions or foster families due to neglect, abuse, or the parents’ inability to provide adequate care. Understanding their status and specific needs is crucial for the development of appropriate support programs.

Gaps in the research stem partly from the fact that, although there is ample empirical evidence of the benefits of physical activity for the general population of children and adolescents, studies examining the impact of sports and recreational activities on OVC populations are virtually nonexistent. This study aims to bridge the gap between knowledge of physical health and dimensions of social inclusion for OVC, highlighting the importance of sports and recreational activities, not only as a tool for physical development but also as a means for the psychosocial empowerment and integration of this population. Precisely due to the lack of research connecting empirical indings with theoretical models of development and learning, this aspect requires systematic analysis and scientiic assessment.

Accordingly, this systematic review aimed to synthesize available evidence on the effects of sports and recreational programs on the physical and psychosocial well-being of children and adolescents without parental care and to delineate speciic circumstances and vulnerabilities of a subgroup within the overarching concept of “vulnerable children.”

## Methods

### Study Design

This study focuses on orphans, children, and adolescents without parental care, 5 to 21 years of age, who are deprived of parental care due to the loss of parents, abandonment, institutionalization, or placement in foster care. These age categories are based on the guidelines of the World Health Organization, according to which “children” refer to the age group of 5 to 12 years, while “adolescents” refer to the age group of 13 to 21 years, taking into account the framework of this study (World Health Organization, 2006).

This systematic review was conducted in accordance with the Preferred Reporting Items for Systematic Reviews and Meta-Analyses (PRISMA) guidelines (Page et al., 2021). The study protocol was registered in the International Database of Prospectively Registered Systematic Reviews in Health and Social Care (PROSPERO CRD420251006123).

### Search Strategy

Two electronic databases, Web of Science and Scopus, were thoroughly searched for all published articles from inception up to June 2024. Keywords were formulated based on expert opinions and relevant systematic reviews in the fi elds of sports management, sports sciences, sociology, inclusive education, social work, and public health, as an interdisciplinary approach is common in research addressing complex issues such as those faced by this vulnerable population. A Boolean search using the operators ‘AND” and “OR” was applied in each database, with the following keywords: (‘physical education’ OR ‘sport’) AND (‘orphans’ OR ‘Orphans and Vulnerable Children’).

### Selection Process

The literature selection was divided into several successive stages: review of titles and abstracts, full-text analysis for eligibility assessment, and evaluation of the methodological quality of the selected studies (risk of bias assessment).

### Eligibility Criteria for Full-Text Review

Studies were assessed for eligibility based on the PICOS criteria (P — Participants, I — Interventions, C — Comparators, O — Outcomes, S — Study Design) (Liberati et al., 2009).

(**P**) The population under investigation included children and adolescents identified as without parental care, with the criteria covering orphans, children who have lost both parents, and children and adolescents placed in residential institutions or foster families due to parental neglect, abuse, or inability to provide adequate care. The study speciically focused on individuals aged 5 to 21 years at the time of data collection. There were no restrictions regarding the demographic characteristics of the studied population. (**I**) The criteria for interventions encompassed the introduction of sports and recreational activities and physical education to the target population. Additionally, observational studies without an intervention, including cross-sectional, longitudinal, and cohort studies, were also considered for inclusion. (**C**) Differences were compared between the experimental group (which participated in the sports and recreational program) and the control group (which was not exposed to this intervention). (**O**) Outcomes measured improvement in psycho-physical health, including indicators such as emotional well-being, stress level, mental stability, physical fitness, and social skills. (**S**) Studies included were longitudinal experimental, longitudinal quasi-experimental, pre-post, prospective longitudinal, as well as cross-sectional studies that did not have an intervention, but provided valuable data for analyzing the current state.

Additionally, studies that were not written in English were also included, if available. Furthermore, unreviewed works (preprints), books, and articles without full text available (only abstracts) were not considered suitable for inclusion in this study. Studies that did not include interventions involving sports and recreational activities did not meet the basic criteria for inclusion in the analysis. In addition, studies were excluded from further review if they included children from impoverished neighborhoods, children and adolescents living on the streets, children in war-torn and post-conflict areas, or if the target population was not clearly defined as orphaned and vulnerable children. Finally, the issues of orphaned and vulnerable children related to comorbidities and the prevalence of chronic diseases caused by HIV infection were not considered within the narrow focus of the research, as the study selection included speciic criteria that did not address this issue.

### Data Extraction

One independent researcher (author) collected all relevant data from the full-text studies. The data were categorized tabularly into the following categories: a) authors and year; b) country; c) study design; d) demographic characteristics of the studied population/sample; e) number of participants, gender, average chronological age; f) follow-up period (if applicable, depending on the study design); g) depending on the study design — characteristics of the population treated with the experimental intervention procedure, treatment method, pre- and post-intervention results for cross-sectional studies, aspects studied through the use of a research tool (questionnaire, etc.) (*Table 1*).

**Table 1:** Main Characteristics of Included Studies

Authors (Year) Country	Design	Sample size, Age	Exp. group	Control Group	Follow-up period	Treated Characteristics	Treatment method/ research tool application	Results
Ismizanov & Rybina (2023) Russia	QES	N= 135.8 21y	N = 22	N= 113	4 years	Psychophysical development, key personal traits (KPT)	Innovative system of physical education and socialization for vulnerable populations	Conclusion of implementation, Targeted orientation Trainer competence Sys. Manageability Pedagogical control
Lazareva & Pristinskii (2008) Russia	QES with pretest and posttest	N = 30, 10-12y	N=15	N=15	2 years	Anti-social behavior, self-confidence, self-aggression, insecurity, aggressiveness, high-risk behaviors	Baseball sections/aggressiveness assessment test (Bass & Darki), self-assessment test (Rubinstein)	Asociality pre 15.2p/postl3.08p. Auto aggression prel8.5p/postl6.2 Self-insecurity prel3.1p/postl2.2 Aggressiveness pre 25.4/post 22p High-risk behaviors pre22.11p/post.19.65p
Tamozhanska etal. (2020) Ukraine	QES with pretest and posttest	N = 28, 5-6y	N=14	N=14	1 year	Physical development, physical preparedness, physical functionality (spirometry)	Moveable games, metered running, breathing exercises	VCL t41.34% RRtl7.18% RVml t40.72% MVRlpm tl9.8% COU2mlpm t23.2 Ht t5.05% Wt t24.66% Stange test t54.12%
Benedikova & Nemcek (2017) Slovakia	Cross-sectional study with one group	N=50, 14.5±2.13y	--------	--------	--------	Popularity of PE classes, Importance of PE, feelings, motivation, preferences in PE classes	Non-standardized questionnaire (Antala, 2012)	Popularity of P.E. Very popular 36% Importance of P.E. Very important 8% Feelings during P.E. Always good 48% Efforts during P.E. Very assiduous 24% Preferences for P.E.
Ngoc & Thuc (2020) Vietnam	QES with pretest and posttest	N=68, lOy	N=30	N=38	3 months	Self-confidence and self-esteem	Athletics, basketball, karate extracurricular activities / Rosenberg Self-Esteem Scale	Self-esteem Low score pre 61.76% post 29.41% Self-confidence Low score pre 64.24%/ post 39.71%
Akhmetshin etal. (2019) Russia	QES with pretest and posttest	N = 20, 14±ly	N = 10	N= 10	7 months	Mental and cultural development, academic achievements (school success)	Section “Football Basics” Self-Development, Cultural and Artistic Events/ TCI-Based Questionnaire (Cloninger, 1994)	Character Traits Exp. Group pre 38 points/ post 63 p. Con. Group pre 35 points/post 44 p. Academic success Exp. Group pre 2.75/post 3.25 Con.Group pre 2.85/2.95

*Note. QES — Quasi-Experimental Study, N — Sample Size, PE — Physical Education, TCI — Temperament and Character Questionnaire, RR — Respiratory rate, VCL — Vital capacity of lungs, RV — Respiratory volume, MVR — Minute volume of respiration, COU2 — Coefficient oxygen utilization in the lungs, Ht — Body height, Wt — Body weight*, Î — *Increase in result values following the intervention or repeated measurement (p <.05)*.

### Risk of Bias Assessment

Due to the characteristic range of study types ultimately included in this systematic review, the authors decided to use the Mixed Methods Appraisal Tool (MMAT) (Hong et al., 2018) for quality appraisal of the included studies. The MMAT consists of a checklist and an explanation of the criteria for each study included. Possible answers to all questions were: “Yes,” “No,” or “Cannot say.” A “No” or “Cannot say” response to two initial questions for study selection on one or both questions from the review section may indicate that the study cannot be assessed using the MMAT. Positive answers to questions related to the assessment of methodological quality indicate high-quality evidence presented in the study, while “No” and “Cannot say” indicate a failure to report precise results that meet the assumptions of the questions. For non-randomized quantitative and descriptive studies, the scoring methodology is presented through the number of criteria met, *i.e*., the number of “Yes” answers concerning the total number of criteria (scores range from 20% — one criterion met to 100% — all criteria met). The quality assessment was independently conducted by the author (P.S.). Any differences in the assessment of study quality were planned to be resolved through discussion, etc.

## Results

### Study Selection

**Figure 1.**
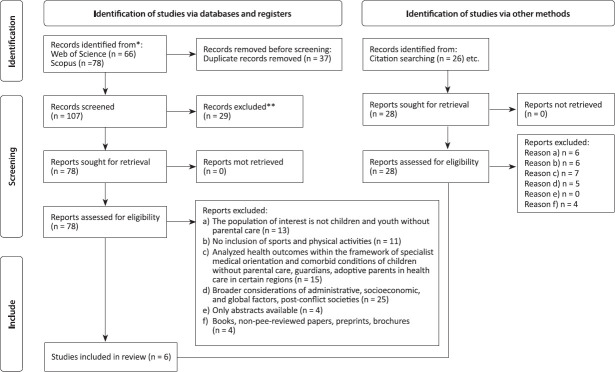
PRISMA Flow Diagram of Preferred Reporting Items for Systematic Reviews and Meta-Analyses illustrates the overall search strategy

The initial search yielded a total of 144 records, with 37 duplicates removed through study analysis. Further, 29 studies were excluded after reviewing the titles and abstracts, and 78 full-text reports were assessed for eligibility based on the inclusion criteria. During the selection process, studies that did not meet the key inclusion criteria were excluded from the analysis. Speciically, studies in which the population of interest was not explicitly deined as OVC were excluded from the analysis. A detailed review of the excluded studies from the initial database search with explanations is presented in Supplementary *File 1*. Based on further searches for relevant citations, 26 new records were identified. The review of excluded studies obtained from references/citations is presented in Supplementary File 2. Finally, after applying all the eligibility criteria, only six studies were included in the analysis: Akhmetshin et al. (2019), Bendíková and Nemcek (2017), Ismiyanov and Rybina (2023), Lazareva and Pristinskii (2008), Ngoc and Th uc (2020), and Tamozhanska et al. (2020). The complete search strategy process is shown in the PRISMA Flow Diagram (*Figure 1*).

### Characteristics of Included Studies

The studies were published from 2008 to 2023. The majority of studies were conducted in Asia, with three originating from Russia (Akhmetshin et al., 2019; Ismiyanov & Rybina, 2023; Lazareva & Pristinskii, 2008), and the other studies conducted in Vietnam (Ngoc & Thuc, 2020), Ukraine (Tamozhanska et al., 2020) and Slovakia (Bendíková & Nemcek, 2017).

The study designs included quasi-experimental studies with pre- and post-intervention measurements (Akhmetshin et al., 2019; Lazareva & Pristinskii, 2008; Ngoc & Thuc, 2020; Tamozhanska et al., 2020), longitudinal studies (Ismiyanov & Rybina, 2023), and cross-sectional studies (Bendíková & Nemcek, 2017). The samples consisted of OVC populations of various ages, including their peers in control groups. The interventions involved physical activities and programs aimed at improving mental and physical health, while control groups typically followed regular activities and were not exposed to the intervention. The measured outcomes included physical itness, stress levels, psychophysical development, self-conidence, and academic achievement, with follow-up periods ranging from 3 months (Ngoc & Thuc, 2020) to 4 years (Ismiyanov & Rybina, 2023), and up to 7 months (Akhmetshin et al., 2019). All studies showed the positive impact of the interventions, particularly in improving physical health and psychosocial well-being, although some had limitations such as small sample size. A total of 331 participants were included in the analyzed studies, with participants’ ages ranging from at least 5 to 21 years. Importantly, according to the ive studies which clearly reported the follow-up period (Akhmetshin et al., 2019; Ismiyanov & Rybina, 2023; Lazareva & Pristinskii, 2008; Ngoc & Thuc, 2020; Tamozhanska et al., 2020), significant results were achieved in the experimental group regarding physical and mental health. All details related to the study design, demographics, and other variables are summarized in *Table 1*.

### Risk of Bias Analysis

As i ve of the studies assessed diferences between the experimental and control groups, they were considered comparative, while the one study that evaluated the attitudes and preferences of children and youth without parental care was categorized as a cross-sectional study (Bendíková & Nemcek, 2017).

The range of total quality scores was between 20% and 60% of the fulfilled criteria out of a total of 100% (each fulfilled criterion carries 20%, with a total of 5 criteria), while the average overall score of the included studies was 46%. Therefore, the quality of each study, as well as the overall quality, was considered moderate. Issues were observed in criteria such as “assessment of confounding factors in the study” and “representativeness of the sample,” while in the cross-sectional study, the criterion “risk of bias due to participant dropout or nonresponse” was negatively evaluated. On the other hand, the highest-rated aspects were related to the “adequacy of measurement concerning outcome/intervention” as well as the “implementation of the intervention within the planned research period” The quality evaluation of the included studies is presented in **Tables 2** and 3 of the paper.

**Table 2:** Mixed Methods Appraisal Tool (MMAT), 2018 version for quantitative non-randomized and descriptive studies

Study Design	Methodological Quality Criteria	1	2	3	4	5	6
Selection Question 1 (all study types)	Are there clear research questions?	Yes	Yes	Yes	Yes	Yes	Yes
	Do the collected data address the research questions?	Yes	Yes	Yes	Yes	Yes	Yes
Quantitative non-randomized studies; Questions	1. Are the participants representative of the target population?	Can’t tell	Can’t tell	Can’t tell	/	Yes	Yes
	2. Are measurements appropriate regarding both the outcome and intervention?	Can’t tell	Yes	Yes	/	Yes	Yes
	3. Are there complete outcome data?	No	Can’t tell	Yes	/	Can’t tell	Can’t tell
	4. Are the confounders accounted for in the design and analysis?	No	No	No	/	No	Can’t tell
	5. During the study period, is the intervention administered (or has exposure occurred) as intended?	Yes	Yes	Yes	/	Yes	Yes
Quantitative descriptive studies; Questions	1. Is the sampling strategy relevant to address the research question?	/	/	/	Yes	/	/
	2. Is the sample representative of the target population?	/	/	/	Can’t tell	/	/
	3 Are the measurements appropriate?	/	/	/	Yes	/	/
	4. Is the risk of nonresponse bias low?	/	/	/	No	/	/
	5. Is the statistical analysis appropriate to answer the research question?	/	/	/	Yes	/	/

*Notes. 1) Ismizanov & Rybina (2023), 2) Lazareva & Pristinskii (2008), 3) Tamozhanska et al. (2020), 4) Benedikova & Nemcek (2017), 5) Ngoc & Thuc (2020), 6) Akhmetshin et al. (2019)*

**Table 3:** Assessment of the Quality of Included Studies/Scores

Included Studies	Final MMAT-Score
Ismizanov & Rybina (2023)	20%
Lazareva & Pristinskii (2008)	40%
Tamozhanska et al. (2020)	60%
Benedikova & Nemcek (2017)	60%
Ngoc & Thuc (2020)	60%
Akhmetshin et al. 2019	60%

### Discussion

A review of the existing research included in this study highlights key trends and gaps in examining combined sports and recreational interventions on the physical and mental health of OVC. In line with the primary objective of this study, one of the most signiicant indings is that interventions in the ield of physical education and sports have shown positive efects in reducing psychological problems (Lazareva & Pristinskii, 2008; Akhmetshin et al., 2019) and improving health status in experimental groups (Tamozhanska et al., 2020), while the results in control groups remained unchanged. In addition to these indings, methodological diferences were identiied, such as varying criteria for defining specificity of populations or interventions, which may hinder direct comparison of results.

Partially in line with the second objective of this study, the findings highlight the speciic circumstances and vulnerabilities of a particular group of children and adolescents within the broader concept of “vulnerable children.” On a global scale, this population numbers 24 million children, with 8-10 million living in orphanages (Wete et al., 2019; Ahern, 2013). Given the large number of OVC, numerous studies indicate that educational and social interventions signiicantly reduce risky behaviors and improve mental health, contributing to their long-term well-being through the development of social skills and provision of tailored support (Akhmetshin et al., 2019; Alizadeh et al., 2020; Bello & Pillay, 2019; Schenk, 2009).

According to Bandura’s social learning theory, the behavior of social workers serves as a model for young people, while group-based upbringing and education enhance adaptability, identiication, and acceptance of problems through shared coping and the realization that they are not alone (Alizadeh et al., 2020). The importance of positive feedback is emphasized, as it increases the sense of competence and self-esteem in adolescents, making them less prone to risky behaviors. One key conclusion is the importance of providing praise and affirmation following the implementation of program activities (Ngoc & Thuc, 2020). This approach can be further understood through the lens of EngestrÖm’s theory of expansive learning, which emphasizes that collaborative activities and cooperation in structured contexts, such as sports and recreational activities, foster the development of new forms of behavior and learning through the transformation of practice and the redefinition of activity goals (EngestrÖm, 1987; EngestrÖm, 2001). In this context, the focus shifts from mistakes and deficiencies to the participants’ potential and progress, with continuous validation from educators or teachers. Emphasis is placed on motivation, regulation of negative emotions, and conscious relection on one’s own development. Such a theoretical framework aligns with EngestrÖm and Sannino’s (2010) view that learning extends beyond knowledge acquisition, encompassing the collective creation of new forms of social practice. This allows for a deeper understanding of the effect of sports and recreation not only on physical development, but also as a social space for learning and empowerment of children without parental care.

The findings of Ngoc and Thuc (2020) are partially consistent with the broader body of literature, which highlights the strong association between participation in structured activities and improved emotional well-being among vulnerable youth. However, despite these positive outcomes, existing evidence also indicates that children and adolescents without parental care remain disproportionately affected by behavioral and emotional problems. Such i ndings can be further understood through the theory of total institutions (Goffman, 1961), which suggests that institutional contexts, such as residential care institutions for children without parental care, shape individuals’ patterns of behavior, identity, and emotional regulation. The closed nature of the system, limited autonomy, and rigid everyday rules may contribute to feelings of depersonalization and reduced self-esteem among children, which helps explain the increased prevalence of behavioral and emotional difficulties within the OVC population. The global prevalence of such difficulties among OVC ranges from 18% to 65% (Kaur et al., 2018). Behavioral and emotional problems are most oten observed in OVC adolescents and male children (Mahanta et al., 2022). In a review study by Kalpana et al. (2020), similar results were found, where OVC were more prone to developing various behavioral issues and mental illnesses such as depression, anxiety, and post-traumatic stress disorder (PTSD). Also, literature shows that physical activities, cultural events, and personal development programs had a signiicant inclusive impact on social development, as well as on academic outcomes for the OVC population (Akhmetshin et al., 2019). The study highlights that OVC participation in sports, cultural, and social activities led to signiicant improvements in social behavior, security in relationships, and academic success. Psychological assessments, measured using Cloninger’s scale, showed a signiicant increase in the experimental group, with scores rising from 4 to 7 (Cloninger, 1994). Academic success also improved from 2.75 to 3.5. These results indicated a positive correlation between physical engagement and educational progress, with improvements in social skills, emotional stability, and reduced anxiety, while the control group showed less progress. Similar indings are reported by the research of Burrus et al. (2018), which discusses educational interventions for vulnerable children, particularly OVC in orphanages, noting improvements in general health.

According to Kalpana (2020) and Kaur et al. (2018), age and gender are signii-cant factors influencing the psychosocial status of OVC. It has been shown that social competency training programs primarily beneit male adolescents living in institutional care (Alizadeh et al., 2020).

The authors emphasize the role of social competence training in reducing high-risk behaviors among male OVC, with a signiicant decrease in scores on the Iranian Adolescents Risk-Taking Scale (IARS; Zadehmohammadi et al., 2011) from 93.9 to 86.81 after the intervention. Similarly, Dang et al. (2017) highlight that social skills training improves the mental and emotional health of at-risk adolescents. Ngoc and Thuc (2020) found that physical education programs, including team sports and group activities, significantly improved self-esteem in children without parental care. Using the Rosenberg Self-Esteem Scale (RSES; Rosenberg, 1989), they observed that before the intervention, 61.76% had low or very low self-esteem (19 very low, 23 low). Afterward, very low self-esteem decreased, and low self-esteem dropped to 15 (39.71%). Statistical analysis confirmed a significant positive impact of physical activities.

The high rating of the importance of physical education (2.72 points) and motivation for team sports and group activities (2.32 points) by children without parental care highlights the need for their social integration (Bendíková & Nemcek, 2017). Comparing these indings with other vulnerable populations, Kurková, Scheetz, and Stelzer (2010) found similar results in children with disabilities, noting that those with psychosensory and motor impairments were also motivated by physical education. Additionally, Kurková, Nemcek, and Labudová (2015) analyzed children with visual impairments and found they preferred individual activities, such as athletics and swimming, to avoid direct contact with others and increase their sense of security. Conversely, children with hearing impairments, as noted by Kurková, Scheetz, and Stelzer (2010), avoided rhythmic activities due to the lack of rhythm-based assis-tive tools. In summary, these indings underscore the importance of inclusive physical activity programs tailored to the speciic vulnerabilities of children and adolescents. They highlight the need for adaptations based on individual needs and comfort levels, supporting the hypothesis that sports-based interventions should address the unique characteristics of each vulnerable population of interest.

Numerous scientiic reports describe malnutrition and neglect of physical health, along with the absence of adequate parental care, as key risk factors contributing to early-age vulnerability among children. This global concern is reflected in child mortality rates — according to the World Health Organization (2016), 15.000 children die every day, amounting to 5.6 million deaths annually (World Health Organization, 2017; UNICEF, 2017a). The primary causes of this high mortality rate include malnutrition, limited access to healthcare, and diseases that are largely preventable with appropriate and timely interventions.

This review shows that OVC face higher rates of malnutrition, poor health, and psychosocial challenges, reflecting global patterns. Studies show that 38.6% of OVCs are malnourished (Mahanta et al., 2022), with rates reaching 57.7% (Reddy et al., 2019) and 60.03% (Chowdhury et al., 2017). Poor nutrition is linked to inadequate caregiver knowledge (Agbozo et al., 2016), and OVCs often report worse physical and mental health than their peers in biological families (Liberati et al., 2009; Patterson et al., 2022).

OVC in institutional care face risks similar to, yet distinctly diferent from, those experienced by children with HIV or those living with HIV-positive adults (Foster, 2006; Sherr et al., 2014). Similar trends have been observed in South Africa, where 3.7 million orphans were recorded in 2016, with over 50% losing their parents due to HIV/AIDS (Bello & Pillay, 2019; UNICEF, 2017a, 2017b). Moreover, in addition to the loss of parents, inadequate nutrition further exacerbates their vulnerability, negatively affecting physical and cognitive development while increasing susceptibility to illnesses and the lack of necessary care. Health risks extend to infectious diseases, particularly HIV. One of the worst-case scenarios is when a child becomes infected with a disease such as HIV at an early age and their parents are HIV-negative. Perinatal HIV leads to severe health decline due to immunodeiciency, with 42% of HIV-infected children in Ukraine growing up in institutions, further affecting their development (Lankford & Higginson, 2020). Given these challenges, studies indicate that structured physical education in treatment and rehabilitation not only addresses psychosocial issues but also has a positive impact on the somatic health of children with compromised well-being (Tarasova & Mankhanov, 2014). Specifically, implementing structured physical education programs has helped reduce the incidence of acute respiratory infections in children born to HIV-infected mothers, decreasing occurrences from 8-10 times to 3-4 times per year (Tamozhanska et al., 2020).

Similarly, the physical health of this population, including oral health, presents a challenge. In Udupi, India, 65% of OVCs sufer from nutrition-related morbidities (Emmanuel & Maheswari, 2017). Dental conditions such as malocclusion (32.5%), calculus (37.3%), gingival recession (13.2%), and decay (19.3%) are common (Ma-hanta et al., 2022). Furthermore, poor nutrition and improper nutrient intake contribute signiicantly to the development of oral diseases, as evidenced by the high prevalence of conditions such as malocclusion, calculus, gingival recession, and decay (Singh et al., 2011). Proper oral hygiene education reduces cavity rates by 40% (Christian et al., 2019), with sugar-induced enamel demineralization, rather than genetics, as a primary cause of caries (Scardina & Messina, 2012; Sheetal et al., 2013). These studies emphasize the importance of education and the link between poor nutrition and oral health in OVCs.

This review highlights the complex challenges of OVC, including psychosocial and health issues. Adapted physical education and empowerment are key to improving their well-being. A comprehensive approach integrating health, education, and psychosocial support is essential, emphasizing the need for ongoing research and tailored community strategies for sustainable solutions.

### Strengths and Limitations

This study has certain strengths that need to be highlighted. To our knowledge, this is the irst systematic review that synthesizes the available scientiic evidence on the impact of physical activities on the physical and mental health of OVC, signiicantly expanding and deepening the current understanding of this issue. Additionally, the results of this study have significant practical implications and provide a valuable contribution primarily to the application of sports and recreation, as well as to all other scientiic disciplines that deal with the study of child and adolescent development. Only studies that met the predefined criteria, including specific criteria, e.g., study type, methodology, sample size, research period, were included in this review. These criteria ensured the inclusion of only relevant and high quality studies focusing spe-ciically on targeted interventions through sports for children without parental care. Another advantage of this study is the expansion of the literature review foundation, which allowed the inclusion of works that were not available in English. Given the limited availability of relevant research in English, the author used translations of works from Russian, enriching the analysis and providing insight into a broader spectrum of knowledge related to the topic, overcoming the language barrier and thus expanding the literature and ofering a new dimension to understanding the problem. Most of the studies covered in this review were published in the last 5 to 8 years. Therefore, the indings and evidence in this review can be considered innovative and original.

However, our study is not without limitations. Speciically, the authors did not take into account the individual case history of the children included in the research. In particular, the status of the population of interest was not clearly deined in terms of the length of stay in the institution, the reasons for entering the institutional care system, or the specific circumstances that led to the loss of parental care or adequate parental support. The absence of these data can significantly impact the representativeness of the sample and the interpretation of the indings. In this regard, it is not methodologically equivalent to analyze the experiences of children who are temporarily placed in an institution due to family crises or short-term interventions, or children placed with foster caregivers temporarily, with children who were abandoned at birth and have grown up in institutional settings from an early age. The lack of diferentiation between these categories may lead to erroneous generalizations, hinder the understanding of speciic developmental outcomes, and reduce the validity of conclusions related to the impact of institutional care on the psychosocial development of children and adolescents.

The heterogeneity of measurement instruments used in the included studies limited the ability to directly compare results and draw firm conclusions about the effectiveness of individual interventions. The reviewed articles employed various tools to assess psychosocial and physical outcomes, often without standardized or validated measures, which may have introduced methodological variability. The diversity of methodologies — ranging from cross-sectional to quasi-experimental and longitudinal designs — and the use of various tools to assess psychosocial and physical outcomes, complicated the direct comparison of results and the synthesis of indings across studies. This methodological variability must be considered when interpreting the conclusions of this review.

In conclusion, all of the studies included in this analysis are deined as studies with moderate overall methodological quality. This highlights the need for further research with more rigorous methodological standards to strengthen the validity of the evidence.

## Conclusion

This systematic review emphasizes the positive impact of targeted interventions through sports and recreational activities on the physical and mental health vulnerabilities of children and youth without parental care. The findings highlight the importance of integrated approaches that address their specific needs, offering valuable insights for relevant scientiic disciplines and stakeholders in child protection and community services. The review underscores the necessity for strategic planning and the development of sustainable solutions within services designed for this population.

Furthermore, the research stresses the need for specialized educational and sports programs in communities and childcare institutions to address health issues, improve social adaptation, and reduce risky behaviors. Public policy plays a key role in managing social innovations and corporate social responsibility, with studies on financial models (Alhazmi et al., 2021; Loren, 2012; Prywes et al., 2004) highlighting the challenges in predicting costs of social services. The involvement of local NGOs and government institutions is crucial for resource allocation and the implementation of innovative approaches (Chimdessa & Cheire, 2018; Abu-Ras et al., 2023).

The findings suggest the need to raise awareness in the corporate sector about volunteerism, social responsibility, and partnerships with local organizations to create sustainable global support models. A multisectoral approach, integrating scientific knowledge, policies, and corporate initiatives, will ensure long-term program stability and better integration of children into society (Ahern, 2013). Future research should also focus on examining gender differences in sports and recreational interventions to reine and improve strategies.
